# Supplementary material for: A Novel Cold-Adapted Nitronate Monooxygenase from Psychrobacter sp. ANT206: Identification, Characterization and Degradation of 2-Nitropropane at Low Temperature
Source: Microorganisms. 2024 Oct 21;12(10):2100. doi: 10.3390/microorganisms12102100 (PMC11510023; doi:10.3390/microorganisms12102100)
Supplement: Supplementary file 1 [file microorganisms-12-02100-s001.zip › microorganisms-3236490-supplementary.pdf]

Supplementary Material

Table S1. Enzyme activity assay and analysis of PsNMO.

Parameter	Volume (mL)	Total protein (mg)	Protein Concentration (mg/mL)	Total enzyme activity (U)	Specific activity (U/mg)	Purification fold	Yield (%)
Crude enzyme	10	87.07	8.71	605.2	6.95	1	100
Purified protein	5	3.19	0.64	310.52	97.34	14	51.31
